# General anesthesia affecting on developing brain: evidence from animal to clinical research

**DOI:** 10.1007/s00540-020-02812-9

**Published:** 2020-06-29

**Authors:** Xinyue Liu, Jing Ji, Guo-Qing Zhao

**Affiliations:** Department of Anesthesiology, China–Japan Union Hospital of Jilin University, Changchun, China

**Keywords:** General anaesthesia, Pre-clinical study, Clinical study, Randomized trial, Neurotoxicity, Cognitive deficit

## Abstract

As the recent update of General anaesthesia compared to spinal anaesthesia (GAS) studies has been published in 2019, together with other clinical evidence, the human studies provided an overwhelming mixed evidence of an association between anaesthesia exposure in early childhood and later neurodevelopment changes in children. Pre-clinical studies in animals provided strong evidence on how anaesthetic and sedative agents (ASAs) causing neurotoxicity in developing brain and deficits in long-term cognitive functions. However pre-clinical results cannot translate to clinical practice directly. Three well designed large population-based human studies strongly indicated that a single brief exposure to general anesthesia (GAs) is not associated with any long-term neurodevelopment deficits in children’s brain. Multiple exposure might cause decrease in processing speed and motor skills of children. However, the association between GAs and neurodevelopment in children is still inconclusive. More clinical studies with larger scale observations, randomized trials with longer duration exposure of GAs and follow-ups, more sensitive outcome measurements, and strict confounder controls are needed in the future to provide more conclusive and informative data. New research area has been developed to contribute in finding solutions for clinical practice as attenuating the neurotoxic effect of ASAs. Xenon and Dexmedetomidine are already used in clinical setting as neuroprotection and anaesthetic sparing-effect, but more research is still needed.

## Introduction

The advent of the modern general anaesthesia (GAs) made it possible for the advancement of modern complex surgical and diagnostic procedures in seriously ill patients of all age groups. Anaesthetic and sedative reagents affect the central nervous system (CNS) by interacting with neurotransmitters and resolving neuronal integration between different brain regions. Presently widely used anesthetics act by two major mechanisms, (1) increasing inhibition through γ-aminobutyric acid (GABA) receptors (e.g., benzodiazepines, barbiturates, propofol, etomidate, isoflurane, enflurane, and halothane) [1] and 2) decreasing the excitation via *N*-methyl-d-asparate (NMDA) receptors [e.g., ketamine, nitrous oxide (N_2_O), and xenon] [2].

In the last 20 years, more and more evidence from animal studies, including non-human primates, have indicated the potentials for GAs agents to cause neuro-apoptosis and other neurodegenerative changes in the developing mammalian brain. It has been demonstrated that exposure to GAs, predominately during the early postnatal period triggers long-term morphological and functional changes in the CNS, which in turn can result in impairment of neurocognitive performance [3]. It arouse the concerns about anaesthesia related neurological injury in young children among parents, health-care providers and regulatory authorities. Several human clinical studies including different outcome measurements interpreted the associations between surgery in early childhood and slightly worse subsequent academic performance or increased risk of the behavioral abnormity. In 2016, the US Food and Drug Administration (FDA) issued a safety announcement, warning that “repeated or lengthy use of general anesthetic and sedation drugs during surgeries or procedures in children younger than 3 years or in pregnant women during their third trimester may affect the development of children’s brains” [4]. And in 2017, FDA issued a change in labeling regarding the safe use of anaesthetic and sedative reagents [5].

As more and more clinical evidence has been revealed about how anaesthetic and sedative reagents affect development of human brain, it is more important to analyze the strengths and limitations of all the clinical evidence in order to determine the changes in clinical practice. This review will be focusing on the new findings in both pre-clinical research and clinical studies, with the brief summarization of the animal studies and current clinical evidence that have been well reported. Finally, the future and ongoing clinical studies will be discussed as the future directions for anaesthetic research on developing brain.

## Pre-clinical evidence: early-life anaesthesia exposure

The initial pre-clinical studies of anaesthesia induced developmental neurotoxicity were done with rodent models. Rodent brains undergo brain growth spurt period (synaptogenesis) mainly at postnatal time. The brain maturation reaches 90–95% of adult weight for fairly short time (postnatal day 20–21). And the key developmental processes, like synaptogenesis, blood–brain barrier establishment and oligodendrocyte maturation etc., across ages are well defined [6, 7]. During normal synaptogenesis, neurons that fail to develop synaptic contact will undergo programmed cell death also known as apoptosis. As for neuronal development, migration, differentiation and synaptogenesis, two of the major factors that influence all are excitatory and inhibitory neurotransmitters. The most important excitatory neurotransmitter that mainly contribute to neurogenesis is glutamate, which acts mainly by activating the NMDA receptor. The most important inhibitory neurotransmitter in mature brain is GABA. In the immature brain, the GABA receptor is excitatory, and its activation leads to a depolarization in the immature neuron. During development, the intracellular chloride concentration decreases, and the GABA receptor is transitioning from being excitatory to inhibitory receptor in the adult brain. Other important factors that involves in neurogenesis are growth factors, like nerve growth factor (NGF) and brain-derived neurotrophic factor (BDNF). These factors mainly regulate the differentiation of progenitor cells, axo- and dendrite-genesis, as well as neuronal cell survival [8].

As known that all commonly used anesthetic and sedative reagents provide anaesthetic and sedative effects by binding to the GABA receptor, or the NMDA receptor, or both. The first animal study by Jevtovic-Todorovic et al., demonstrated that routine GAs (isoflurane, sevoflurane, propofol, ketamine) are capable of producing lasting cognitive, behavioral and memory deficiency in postnatal day 7 rats when exposed with 6 h mixture of nitro oxide, isoflurane and midazolam [2]. More cellular and animal studies have provided substantial and convincing evidence on the cytotoxic and neurotoxic effects of GAs. Furthermore, studies on non-human primates aligned with the results in rodents research, finding that early-life exposure to ketamine, sevoflurane or isoflurane can lead to persistent decline in cognitive, executive, memory and motivation-based tasks, and increase anxiety behaviours in long term [9–11].

The molecular mechanisms of cytotoxic and neurotoxic effect of general anaesthetics on developing brain have been extensively explored. In-vitro and in-vivo studies have revealed that anaesthetics induce apoptosis via two possible pathways: intrinsic pathway and extrinsic pathway [12]. The extrinsic pathway is activated via tumour necrosis factor (TNF) receptors. While the intrinsic pathway is initiated in response to signals from within the cell, this results in the decreasing anti-apoptotic BCL-2/pro-apoptotic Bax ratio, increasing reactive oxygen species (ROS), and promoting cytochrome C to be released from the mitochondria and activating caspase-3 cleavage. GAs accelerates the process of apoptosis during the period when GABA receptor is excitatory. While applying the GAs at a later neuronal development stage when GABA receptor is inhibitory, it induces less neurodegeneration. However, the negative effects on learning and memory are still exist. Both long-term blockage of excitatory and the activation of inhibitory receptors, neuronal synapses may induce a change in receptor expression. This might influence the excitability of the neuron and make it more vulnerable to toxic stimuli. For neuronal development, both excitatory and inhibitory input from adjacent neurons are essential. By blocking the connection from adjacent neurons, the differentiation and survival might be critically affected. Other studies also suggested that GAs causes deficit in axon myelination by affecting glial cells [13]. Recently anaesthetic-induced neuroinflammation has been revealed as a possible mechanism for cognitive impairment in immature mice [14].

The current available animal studies give rises to several important indications of factors that affect toxicity of early-life anaesthesia on animals. First is the developmental stage at which animals are exposed to GAs. Neuronal cells tend to be more vulnerable during the brain growth spurt period. And the timing of this period varies from species, in rat, it lasts from the 7th up to 17th postnatal day and in rhesus monkeys from the 5th to the 16th postnatal day [6]. The peak period of synaptogenesis various between brain regions and different neuronal cell types [15]. Different neuronal cell types may possess different susceptibility to GAs. Glutaminergic and GABAergic neurons may be more vulnerable to toxic effects than cholinergic neurons [16].

Second important factor is the length and frequency of exposure to the general anaesthetics. Neurocognitive deficits have been identified after several hours GAs administration but not after short-term single administration of GAs [17]. Repeated, short-time exposures to the GAs also results in cognitive dysfunction, indicating that accumulative length of exposure to GAs within a certain period of time is one of key factors [14, 18].

Third, a dose-dependent toxicity effect has been revealed in animal studies. Higher the dose of GAs is, the larger the number of apoptotic neurons is. The dosage of GAs can affect the degree of developmental impairment, cell differentiation and synaptogenesis [1, 19].

Lastly, recent studies suggest that the sensitivity to GAs may vary between sexes. One study in rats showed males and females followed distinct paths of neural and cognitive development after an early anesthetic-mediated effect on the brain. Both male and female rats exhibited extensive neuronal death. However drastic behavioral impairment was manifested only in male subjects [20].

## The gap and limitation of the findings from animal studies transferring to clinical practice

Researchers have focused substantial attention to evaluating the development of cognitive abilities of animals exposed to GAs at the peak of synaptogenesis. It has been concluded that GAs exposure animals showed abnormality in memory and cognitive in adulthood comparing with GAs non-exposure animals. Both single long exposure and repeatedly, short-term exposure to GAs during critical stage of brain development can cause significant impairments in neurocognitive development. We know that the critical stages of brain development various from species. Then how to imply the findings from animal research to clinical practice?

In human brain, the synaptogenesis period is thought to last from the last trimester until the third year of life which is way longer comparing to rodent and rhesus monkeys. It is hard to imply the critical development stages that mostly affected by GAs directly from animal studies [21]. Besides, the animal studies basically used healthy animals to expose to GAs. In the clinical situation, it is often with children in need of surgery, whom might be exposed to prolonged surgeries and have multiple postoperative complications, like pain, anxiety, fluid imbalance, and surgery-induced trauma [22]. There are only limited animal studies including surgical or pain stimuli into consideration, but the results are contradictory. One study published in 2012 by Shu et al., showed that rat pups who received GAs for 6 h with a hind paw incision or formalin injection respectively, exhibited higher degree of neuro-apoptosis in brain cortex and spinal cord. These subjects also showed long-term impairments comparing to age-matched animals which have been exposed to GAs alone without nociceptive stimulus [23]. Interestingly another study published in the same year by Liu et al., suggested that 6 h exposure of rat pups to GAs with chemical nociception induced by complete Freund’s adjuvant resulted in attenuated anaesthesia-induced neuro-apoptotic response. Cognitive behavior in later life was not assessed [24]. All these indicating that the real situation in clinical setting is way more complicated. It would be challenging to establish a clear relevance of the animal studies to clinical practice. However pre-clinical animal studies are still valuable as they revealed the molecular and cellular mechanism for GAs toxicity in developing brain [25]. The behavior and pathological data from animal studies can provide reference for the designing clinical studies.

## Clinical evidence: what we can imply from all the clinical data and what’s next

The first reported study in 1953 by Eckenhoff et al., suggested possible relevance between personality change and pre-anaesthetic medication (pentobarbital, scopolamine and morphine) [26]. However no clear link between surgery and neurodevelopmental outcome had been noticed outside the neonatal period. There was no obvious clinical problem, until the pre-clinical data has grown. There was an increasing urge to find out if GAs exposure does indeed cause the clinical relevance of neurodevelopment dysfunction in children. The answer to this question is not easy or straightforward. There are many aspects and concerns needed to be taken into consideration, regarding the GAs effects on children. The effect might be dependent on the choice of anaesthetics. The GAs approach varies based on the child’s age, medical/surgical history, types of surgical procedure and duration of anaesthesia administration and the nature of the anaesthesia exposure. Unfortunately, translation pre-clinical data in human is imprecise and inapplicable. A recent review by integrating more than 440 pre-clinical studies with exceed 30 clinical studies up to date, demonstrated no clear exposure duration threshold below which no structural injury or subsequent cognitive abnormalities occurred. Animal data did not clearly identify a specific age beyond which anaesthetic exposure did not cause any structural or functional abnormalities [27]. All these make it even harder for human studies to use pre-clinical data as a guideline to design clinical research.

Given the uncertainty of translating the pre-clinical data, it is impossible to design a single randomized controlled human study including all the affecting factors to determine the outcomes. It is more appropriate for a range of studies to examine a range of outcomes, anaesthetic duration and age at exposure. Many elaborate reviews on this topic have been published recently and assessed most of the clinical studies [3, 28–31]. In this review, we will briefly summarize the up-to-date and the most well designed three clinical studies and discuss the limitations. Most importantly, we will discuss the implications from human studies that can guide the clinical practice and future directions for designing clinical research.

## The Pediatric Anaesthesia Neurodevelopment Assessment (PANDA) Study

PANDA study used a sibling-matched cohort design to test if a single anaesthesia exposure in healthy young children is associated with impaired neurocognitive development and abnormal behavior in later children. The study cohort included sibling pairs within 36 months in age and currently 8–15 years old. All exposed children received inhaled anaesthetic agents and anaesthesia median duration of 80 min. 105 sibling pairs were included in the primary outcome, global cognitive function (IQ) test. There was no statistically significantly differences in mean scores between exposed siblings and unexposed siblings. A detailed neuropsychological battery assessed IQ and domain-specific neurocognitive functions as the secondary outcomes also showed no statistically significant differences between sibling pairs in memory/learning, motor/processing speed, visuospatial function, attention, executive function, language, or behavior [32].

## The general anaesthesia compared to spinal anaesthesia (GAS) study

The first randomised trial was designed as GAS consortium to verify whether anaesthesia exposure in early childhood can cause long-term neurodevelopment changes. Two established anaesthetic techniques for inguinal herniorrhaphy treating postoperative apnea in young infant: awake-regional and sevoflurane-based Gas [33]. Between the year 2007–2013, 722 infants up to 60 weeks who were recruited in the trial from 28 hospitals in multiple countries were randomly assigned to receive either awake-regional or sevoflurane-based GAs for inguinal herniorrhaphy. The median duration of anaesthesia in the GAs group was 54 min. The outcomes of neurodevelopment were assessed at 2 years of age and 5 years of age respectively. The secondary outcome, the composite cognitive score of the Bayley Scales of Infant and Toddler Development III, assessed at 2 years of age and published in 2016, providing strong evidence for equivalence between awake-regional anaesthesia and GAs in infancy in terms of neurodevelopment [34]. The primary outcome measure was full-scale intelligence quotient (FSIQ) on the Wechsler Preschool and Primary Scale of Intelligence, third edition at 5 years of age published recently in 2019. The results were interpreted that slightly less than 1 h of GAs in early infancy does not alter neurodevelopmental outcome at 5 years of age compared with awake-regional anaesthesia in a predominantly male study population [35].

## Mayo Anesthesia Safety in Kids (MASK) study

MASK study enrolled children born in Olmsted County, Minnesota, USA, from 1994 to 2007, who were exposed to surgery and anaesthesia before the age of 3 year. Total 997 children were enrolled in the MASK study. 380 children had a single exposure to GAs, 206 children had multiple exposure to GAs, and 411 were unexposed. Enrolled children were sampled using a propensity-guided approach and underwent neuropsychological testing at age 8–12 or 15–20 year. The primary outcome based on the Full-Scale intelligence quotient standard score of the Wechsler Abbreviated Scale of Intelligence did not differ significantly according to exposure status. Secondary assessment including individual domains from a comprehensive neuropsychological assessment and parent reports, found that processing speed and fine motor abilities were decreased in the multiple but not the single exposed children. Other functions did not show significant difference accordingly. The parents of multiple exposed children reported increased problems related to executive function, behaviour, and reading [36, 37].

Two follow-up studies of MASK study attempted to translate the non-human primate data to humans and perform additional analysis to verify the results [38, 39]. One follow-up study done by Warner et al., proposed reasons for the variability in the results of anaesthetics clinical neurotoxicity. As with neurotoxic exposure, not all cognitive domains might be equally affected and specific domains that are affected by GAs are still not clear. By applying the knowledge from animal studies, Warner and colleagues picked the neurodevelopment test, Operant Test Battery (OTB) which can be used both in humans and animals. There was no difference found in OTB scores even after multiple exposure in children. However, previous non-human primate studies showed decrease in OTB accuracy and response speed after anaesthetic exposure [11]. The difference of the findings might be due to the insensitivity of OTB test for detecting small effects in children. Also the median anaesthetic exposure duration for children in MASK study was 45 min for single exposure and 187 min for multiple exposure [36], while the non-human primate study used a 24 h infusion of ketamine, which is not only a very long exposure but also an anaesthetic drug that is not the choice in most paediatric procedures [11]. This MASK study finding is consistent with other published studies showing that anaesthetic exposure does not result in large cognitive deficits, particularly in domains associated with intelligence.

The other MASK follow-up study done by Zaccariello et al., preformed a secondary re-analysis of some of the neuropsychologist-assessed outcomes presented in the initial MASK study [36]. Two analyses, a factor and a cluster analysis were performed in this follow-up study. The results showed that children with multiple exposures had lower scores in processing speed, motor coordination and visual-motor integration, but no differences were seen in the children after a single exposure [39].

GAS and PANDA studies provided strong evidence that infants exposing to a single brief (less than 1 h) GAs does not cause significant neurocognitive or behavioural deficits. The MASK study also verified the same conclusion. Besides, MASK study provided evidence that not the single exposure but the multiple exposure to GAs in young children is associated with a specific pattern of deficits. However, this is just the beginning of understanding how GAs affects neurodevelopment in infants. The current published clinical studies have limitations to address the issue thoroughly and there are still missing puzzles.

First of all, the dosage dependent effect of GAs is still not clear. GAS and PANDA studies all used less than 1 h brief anaesthetic exposure showing no effect in long-term neurodevelopment. MASK studies indicated that single average 45 min exposure does not affect gross neurodevelopment, while multiple exposure with average of 187 min period caused the deficits in fine motor skills. Current results do not provide data regarding to the neurocognitive risks of repeated episodes of anesthesia exposure or more prolonged durations of a single exposure which often happen in complicated pediatric surgeries.

Secondly, the current cognitive and behavioral tests used for clinical studies are not sensitive enough to analysis in depth how affected sub-domains in the brain are. Like the Bayley III assessment method used in GAS studies is a well validated assessment for current neurodevelopment and early neurobehavioral assessment of children. However, it is not a perfect predictor for long-term outcome. Further analysis of the data collected from current clinical studies could also provide more information and evidence [40].

Thirdly, the GAs effect on different sex has not been fully addressed in the PANDA and GAS study. Pre-clinical data indicated that anaesthetic drugs might affect long-term cognitive function differently between sexes [20]. Only MASK studies females and males were equally recruited in the studies, but not separately analyzed. In PANDA and GAS studies, the majority of the recruited children were males [27].

Last but not least, all three studies were assessed the association between surgery plus anesthetic exposure and cognitive/behavior deficiency. It is hard to rule out the impact of the surgery contribution to the outcomes. Furthermore, confounders such as hypotension, body temperature, and hypoxia during surgery are rarely included in these studies. These confounders could potentially alter the outcomes [30].

## Discussions and conclusions

In pre-clinical studies, adverse neurological effects of commonly used anaesthetic agents are seen in rodents and non-human primates. Long duration, repeated exposure and multiple agents can increase neurotoxicity, cognitive and behavioral function. Due to the difference in brain development between human and other animals, the results of the pre-clinical studies cannot directly apply to clinical practice. SmartTots, a public–private partnership (PPP) was established between the FDA and the International Anesthesia Research Society (IARS) in 2009, to facilitate pediatric anesthesia research with aim of making surgery safer for infants and children [41, 42]. Multiple published epidemiologic studies assessed the association between GAs exposure in early life and neurodevelopment in later life of children. Due to the limitation of the sample size and lack of sensitivity of the measurements, the results showed various outcomes and some contradictories [29, 43–45]. Three well-designed clinical studies discussed in this review found evidence showing that not a single brief exposure but multiple exposure to GAs in children within 3 years age decreased processing speed and motor skills. The difference was not found in other cognitive functions [32, 34–36, 46]. One of the MASK follow-up studies provided more information and possible future directions for further designing clinical studies. The results showed that anaesthetic exposure, even multiple exposure, did not result in gross deficits in all exposed children. This indicates that the effects of GAs on children are likely to be subtle and not all children are universally vulnerable [39]. As there are important limitations in the published observational studies, it is still not sufficient in evidence to conclude that GAs directly has any long-term impact in children [47]. Besides more clinical studies with larger scale observation in need for sequential clinical studies. Randomized trials with longer duration exposure of GAs, follow-ups, more sensitive outcome measurements, and strict confounder controls should also be included to provide more conclusive and informative data. Meanwhile, new research area has been developed in order to find possible solutions that can attenuate anaesthetics neurotoxicity effect on developing brain. Different drugs are being studied to mitigate the apoptosis response to ASAs. Xenon and Dexmedetomidine (DEX) are already used in clinical settings as neuroprotection and anaesthetic sparing-effect, but more research is still needed [48–51].

Evidence from clinical studies on association between GAs and neurodevelopment provided more information for clinical practice. While, at this point it is still inconclusive. Thus, changes in clinical management of children are not advised at this time. It is less important than the benefit of medical procedure has done for the children. However, in order to allow parents and healthcare providers to make informed clinical decision, the risk of anaesthesia should be adequately and fully evaluated.

## Abbreviations

GAs: General anaesthesia
GABA: γ-Aminobutyric acid
NMDA: *N*-Methyl-d-asparate
CNS: Central nerve system
FDA: US Food and Drug Administration
NGF: Nerve growth factor
TNF: Tumour necrosis factor
ROS: Reactive oxygen species
PANDA: Pediatric Anaesthesia Neurodevelopment Assessment
GAS: General anaesthesia compared to spinal anaesthesia
MASK: Mayo Anesthesia Safety in Kids
FSIQ: Full-scale intelligence quotient
IQ: Cognitive function
OTB: Operant Test Battery
PPP: Public–private partnership
ASAs: Anaesthetic and sedative agents
